# A Decomposition-Based Evolutionary Algorithm with Neighborhood Region Domination

**DOI:** 10.3390/biomimetics10010019

**Published:** 2025-01-02

**Authors:** Hongfeng Ma, Jiaxu Ning, Jie Zheng, Changsheng Zhang

**Affiliations:** 1 School of Information Science and Engineering, Shenyang Ligong University, Shenyang 110159, China; mahongfeng@stu.sylu.edu.cn (H.M.); zhengjie@stu.sylu.edu.cn (J.Z.); 2 Software College of Northeastern University, Northeastern University, Shenyang 110819, China; zhangchangsheng@mail.neu.edu.cn

**Keywords:** MOEA/D, neighborhood, neighborhood region domination, ideal point, intelligence techniques

## Abstract

The decomposition-based multi-objective optimization algorithm MOEA/D (multi-objective evolutionary algorithm based on decomposition) introduces the concept of neighborhood, where each sub-problem requires optimization through solutions within its neighborhood. Due to the comparison being only with solutions in the neighborhood, the obtained set of solutions is not sufficiently diverse, leading to poorer convergence properties. In order to adequately acquire a high-quality set of solutions, this algorithm requires a large number of population iterations, which in turn results in relatively low computational efficiency. To address this issue, this paper proposes an algorithm termed MOEA/D-NRD, which is based on neighborhood region domination in the MOEA/D framework. In the improved algorithm, domination relationships are determined by comparing offspring solutions against neighborhood ideal points and neighborhood worst points. By selecting appropriate solution sets within these comparison regions, the solution sets can approach the ideal points more and faster, thereby accelerating population convergence and enhancing the computational efficiency of the algorithm.

## 1. Introduction

Over past decades, researchers have proposed various multi-objective evolutionary algorithms (MOEAs) to solve multi-objective optimization problems (MOPs). The NSGA-series algorithms, based on Pareto dominance relationships, have evolved from the initial NSGA-II to the latest NSGA-IV, continuously improving algorithm performance through enhanced selection mechanisms and diversity maintenance strategies [1,2]. The strength Pareto evolutionary algorithm (SPEA2) has demonstrated excellent performance in maintaining population diversity through precise fitness assignment and density estimation schemes [3].

Beyond traditional evolutionary algorithms, researchers have extended other intelligent optimization techniques to the multi-objective domain. Multi-objective particle swarm optimization (MOPSO) achieves effective searching in the objective space by introducing external archive mechanisms and leader selection strategies [4]. Inspired by grey wolf pack behavior, multi-objective grey wolf optimizer (MOGWO) employs hierarchical structures and prey-encircling mechanisms, showing unique advantages in solving complex multi-objective problems [5].

Although these algorithms have demonstrated their respective advantages in different application scenarios, they still face challenges when dealing with problems having complex Pareto fronts. The decomposition-based multi-objective evolutionary algorithm MOEA/D offers a new perspective, where the concept of neighborhood is introduced [6]. Specifically, the MOEA/D method decomposes the multi-objective optimization problem into multiple subproblems. For each decomposed subproblem, there is a specific weight vector.

In the MOEA/D algorithm, crossover and mutation operations within the same neighborhood are more likely to produce superior offspring. The generated offspring then replace their parent solutions in the neighborhood that they dominate, thereby accelerating the convergence of the population.

**Figure 1:**
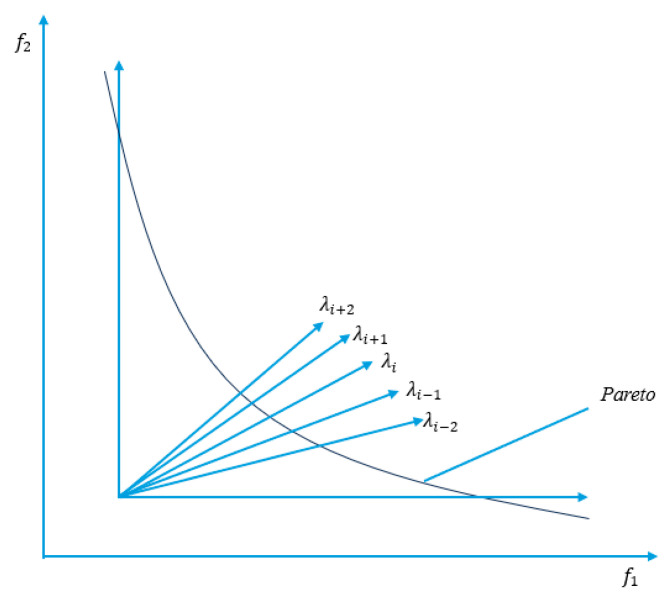
MOEA/D neighbor diagram.

To illustrate this concept with an example, as depicted in Figure 1, when T equals 3, for a given $\lambda_{i}$, its neighborhood would include $\lambda_{i+1}$, $\lambda_{i}$, and $\lambda_{i-1}$. Similarly, for $\lambda_{i+1}$, its neighborhood would consist of $\lambda_{i+2}$, $\lambda_{i+1}$, and $\lambda_{i}$. This setup encourages local competition and collaboration among neighboring solutions, leading to more effective exploration of the Pareto front while maintaining diversity within the population.

In terms of solution quality, both the MOEA/D and NSGA-II algorithms effectively approximate the Pareto front. However, when it comes to speed, MOEA/D [6] performs significantly faster on average compared to NSGA-II [1] in benchmark tests. For biobjective test suites, MOEA/D’s average running time is approximately twice as fast as NSGA-II. In the case of triobjective test sets, MOEA/D’s average speed surpasses that of NSGA-II by more than eightfold [6].

In MOEA/D, the quality of solutions generated during the crossover and mutation process is often uneven, wherein the purpose of the newly generated offspring is to update the individuals within their respective neighborhoods. Superior offspring have an impact that often extends beyond their immediate neighborhood, potentially influencing the entire population. However, the MOEA/D algorithm does not employ a separate operation targeted at these individuals to facilitate their swift exploitation within the population or to enhance their utilization rate within the population. The traditional MOEA/D algorithm requires multiple iterations before superior offspring can gradually spread and impact their entire search region. Meanwhile, for the inferior-quality offspring, undergoing the process of updating their neighborhood individuals not only hinders convergence but also degrades the overall quality of the solution set. These inferior offspring often contribute little value to their neighborhoods or the population at large, yet conventional MOEA/D lacks a process to screen these offspring individuals. To address this, we propose a neighborhood region domination strategy that filters the generated offspring to enhance the utilization of superior solutions and discard inferior ones, thereby improving the algorithm’s convergence and performance.

Firstly, our strategy introduces two points: the neighborhood ideal point and the neighborhood worst point. The neighborhood ideal point dominates all individuals within the neighborhood, while the neighborhood worst point is dominated by every individual in the neighborhood. Therefore, these two points collectively provide a good representation of the information within the neighborhood. For superior offspring, they should dominate all individuals in the neighborhood. Consequently, we only need to compare them with the neighborhood ideal point to ascertain their dominance relationship. If the offspring dominates the neighborhood ideal point, its reference for update shifts from the neighborhood to the entire population. This approach swiftly harnesses these superior offspring, enabling the algorithm to converge more rapidly toward the Pareto front.

Regarding the generation of some relatively inferior offspring, we compare them with the neighborhood worst point. If the neighborhood worst point dominates these offspring, it suggests that such new offspring do not contribute any significant reference value to the neighborhood. Consequently, we refrain from incorporating these offspring into the population for updating individuals, effectively preventing a decline in population quality due to their introduction.

Our strategy facilitates the swift exploitation of superior offspring while setting a threshold for offspring solutions to prevent inferior ones from deteriorating the overall population quality, thereby enhancing the algorithm’s convergence and performance. This paper incorporates this strategy into the MOEA/D algorithm, proposing a multi-objective evolutionary algorithm based on neighborhood region domination (MOEA/D-NRD). This algorithm determines the domination relationship between offspring solutions and their neighborhood’s ideal point as well as the neighborhood worst point. By using such comparisons within specifically defined neighborhood regions, the algorithm efficiently guides the solution set to approach the ideal point more extensively and rapidly, thereby accelerating the convergence of the population.

## 2. Related Work

### 2.1. MOPs

The general form of a multi-objective problem (MOP) can be represented as:

(1) $$Minimize\;F(x)={\left(f_{1}\left(x\right),f_{2}\left(x\right),\cdots ,f_{m}\left(x\right)\right)}^{T}\;s.t.\left\{\begin{array}{c} h_{i}\left(x\right)=0,\;\;i=1,\cdots ,n_{h}\\ g_{i}\left(x\right)\leq 0,\;\;i=1,\cdots ,n_{g}\\ x\in \Omega_{x} \end{array}\right.$$

where m represents the number of objective functions, $n_{h}$ and $n_{g}$ denote the number of equality and inequality constraints, respectively, $\Omega_{x}\subseteq R^{n}$ denotes the decision space, and $F(x):\Omega_{x}\to R^{m}$ is the vector of objective functions for solution x.

In comparison to single-objective optimization, multi-objective optimization encounters inherent conflicts among objectives, leading to a situation where optimizing one objective can result in the degradation of other objectives. Consequently, it is difficult to identify a unique optimal solution. In multi-objective optimization, the aim is often to achieve the best overall compromise among multiple objectives. Below are some definitions related to the Pareto concept.

1. Pareto dominance

If and only if $\forall f_{t}(x_{i})\leq \;f_{t}(x_{j})$, $\exists f_{t}(x_{i})<\;f_{t}(x_{j})$, and j=1,2,…,m, a solution $x_{i}$ is said to dominate $x_{j}$, denoted as $x_{i}≺x_{j}$.

2. Pareto optimal

The solution $x^{*}$ is considered optimal in the decision space if and only if there does not exist another solution $x\in \Omega_{x}$ such that $F(x)\leq \;F(x^{*})$, with at least one objective function satisfying $f_{i}(x)\leq \;f_{i}(x^{*})$.

3. Pareto set (PS)

For a multi-objective optimization problem, given a set of optimal solutions, if the solutions within this set are non-dominated with respect to each other, meaning that no solution dominates any other in the set, then this set is referred to as the Pareto set.

4. Pareto front (PF)

The set of objective function value vectors corresponding to each solution in the Pareto set is referred to as the Pareto front.

Multi-objective optimization plays a pivotal role in addressing real-life problems that inherently involve multiple, often conflicting objectives. For instance, in urban planning, optimizing traffic flow and reducing pollution levels simultaneously requires a multi-objective approach due to the inherent trade-offs between maximizing vehicle throughput and minimizing emissions. Another example lies in financial portfolio management, where investors seek to maximize returns while minimizing risk, leading to a natural application of multi-objective optimization techniques.

### 2.2. Evolution of MOEA

The evolution of multi-objective optimization algorithms has been significantly influenced by advances in genetic algorithm implementations, particularly through real-coded genetic algorithms (RCGAs) and PSO-based techniques. Unlike traditional binary-coded genetic algorithms, RCGAs were specifically designed to handle continuous optimization problems more effectively. In 1998, Herrera established the fundamental framework for RCGAs by introducing specialized genetic operators that worked directly in the real number space, marking a significant advancement in evolutionary computation [7]. Coello, Coello, and Lechuga proposed MOPSO in 2002, the first comprehensive adaptation of PSO for multi-objective optimization, incorporating an external archive for storing non-dominated solutions and introducing novel leader-selection mechanisms [4]. In 2005, this work was further refined by Reyes-Sierra with oMOPSO, which introduced ε-dominance concepts and multiple mutation operators to enhance the algorithm’s exploration capabilities [8]. In 2009, the evolution of PSO-based approaches reached a notable milestone with SMPSO, developed by Nebro, which addressed the velocity explosion issues in previous PSO variants through an effective velocity constriction mechanism [9]. In 2020, Akopov designed a parallel real-coded genetic algorithm (P-RCGA) for the cluster-based optimization of an evacuation process [10]. In 2022, Kumar combined particle swarm optimization with crowding distance to propose MOPSO-CD, which was applied to solve the reliability-cost frontiers in complex network bridge systems [11].

The relative success and limitations of these approaches significantly influenced the development of decomposition-based methods, particularly MOEA/D, which would later emerge as a powerful framework for multi-objective optimization.

### 2.3. MOEA/D

The decomposition-based multi-objective algorithm MOEA/D, proposed by Zhang and Li, diverges from the traditional method of assessing solution quality based on domination relationships. Instead, it uses a set of predefined weight vectors to uniformly partition the entire objective space into multiple search directions or subproblems. Each weight vector designates a unique direction for the algorithm to explore, allowing for the definition of single-objective or simplified multi-objective subproblems for each search orientation.

In MOEA/D, for every search direction, there are T pre-assigned nearest neighbors, which facilitate an efficient and cooperative approach to solving the corresponding single-objective problem. During the search process, mating selection and replacement decisions take into account solutions related to T-neighboring search directions.

MOEA/D operates as a steady-state algorithm, incrementally updating its solutions over time, thereby approximating the true Pareto-optimal front in a stepwise manner. This iterative updating process allows the algorithm to maintain a balance between convergence toward the optimal solutions and maintaining diversity within the population, thus improving overall performance in addressing complex multi-objective optimization problems.

### 2.4. MOEA/D Variants

The MOEA/D algorithm was initially proposed by Zhang and Li in 2007 [6], and since then has been widely applied and further refined in subsequent research. The fundamental idea behind the MOEA/D algorithm is to decompose a multi-objective problem into N scalar optimization subproblems and then use evolutionary algorithms to optimize the solution sets for each individual sub-problem. By introducing the concept of neighborhood, MOEA/D is able to identify a set of non-dominated solutions within each subproblem’s solution set, thereby leading to the overall collection of non-dominated solutions for the entire multi-objective problem. However, the neighborhood strategy in the original version of MOEA/D is not without its imperfections. In 2009, Zhang pointed out that unlimited neighborhood updating can lead to a loss of population diversity during the evolutionary process [12]. In the same year, Zhang proposed the MOEA/D-DRA algorithm, which introduced a dynamic resource allocation strategy. This strategy adjusts reference points and their corresponding resource allocations according to the evolutionary stage of the population, effectively enhancing the algorithm’s convergence and overall performance [13]. In 2012, Zhao et al. proposed the ENS-MOEA/D, which used an ensemble of different NSs of the subproblems with online self-adaptation. Through experimental analysis, they investigated the impact of neighborhood size on MOEA/D’s performance, noting that different problems might be better suited to different neighborhood size settings. This algorithm dynamically adjusts the neighborhood size based on the current state of the optimization process [14]. In 2014, Liu proposed ADEMO/D-ENS, employing a variable neighborhood decomposition-based method for optimization [15]. Wang proposed the MOEA/D-AGR, which integrates an adaptive global replacement strategy into the neighborhood updating mechanism. This addresses the shortcomings in traditional neighborhood update strategies concerning global searches and facilitates the discovery of a greater number of elite solutions. Wang introduced the MOEA/D-AGR, incorporating an adaptive global replacement strategy into its neighborhood updating mechanism. This innovation addresses the deficiency in traditional neighborhood update strategies regarding their exploration of the global search space [16]. Li proposed MOEA/D-STM, which incorporates the stable matching model into the MOEA/D algorithm, leveraging the stable matching model to address the issue of maintaining diversity within the population [17]. In 2016, Zhang proposed the MOEA/D-GR algorithm, which dynamically adjusts the neighborhood size based on the population’s distribution characteristics and convergence status. This method implements the global replacement of elite solutions, effectively maintaining the diversity of the population and thereby enhancing the algorithm’s adaptability to complex multi-objective problems [18]. In 2019, Wang proposed the MOEA/D-ANS algorithm, which adaptively adjusts the neighborhood size based on the overall evolutionary progress of the population and the specific advancements in each sub-problem. This approach aims to strike a balance between the convergence and distribution of the population [19]. Fan proposed MOEA/D-IEpsilon, an algorithm that dynamically adjusts the constraint-handling method based on the ratio of feasible solutions to the total solutions in the current population [20]. In 2021, Xu and Zhang improved upon the dynamic resource allocation strategy in MOEA/D-DRA by proposing an enhanced algorithm called MOEA/D-ANA, which adaptively adjusts neighborhood sizes. This modification controls the solution density of subproblems, thereby enhancing population diversity [21].

## 3. MOEA/D-NRD

### 3.1. The Fundamental Idea

For MOEA/D, its fundamental idea revolves around iteratively optimizing a population over multiple rounds to gradually approach the ideal Pareto front. In the solving process, offspring solutions are assessed by comparing only against their neighborhood solutions, which can lead to insufficient exploration and poor convergence properties of the solution set. To overcome this issue and adequately obtain a high-quality set of solutions, the algorithm requires a large number of iterations, thus lowering its computational efficiency.

In the improved algorithm, to solve this problem, offspring positions are generated and their quality is predicted in advance, which enables the selection of appropriate reference populations for them. This neighborhood region domination strategy ensures both the convergence of the algorithm and enhances its computational efficiency, assuming that the multi-objective optimization problem addressed in this context involves a biobjective minimization problem.

**Figure 2:**
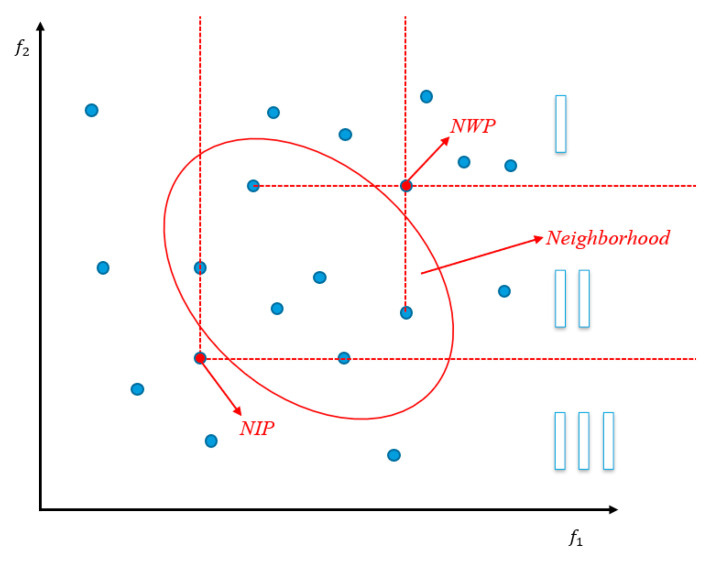
Schematic diagram of dynamic selection strategy.

As shown in Figure 2, the neighborhood region domination strategy divides the entire potential offspring generation area into three parts based on the ideal point, the neighborhood ideal point, and the neighborhood worst point. The ideal point represents a point composed of the minimum values achieved by the current population across all objectives. Denoting $z_{i}^{*}$ as the i-th subobjective of the ideal point, its definition is given as follows:

(2) $$z_{i}^{*}=min\;\{f_{i}(x)|i=1,2,\ldots ,m,\;x\epsilon \Omega \}$$

The neighborhood ideal point (NIP) represents a point composed of the minimum values achieved by the neighborhood population from which the offspring’s parents are drawn for each objective. In this context, let ${z(NIP)}_{i}^{*}$ denote the i-th subobjective of the neighborhood ideal point; its definition is as follows:

(3) $${z(NIP)}_{i}^{*}=min\;\{f_{i}(x)|i=1,2,\ldots ,m,\;x\epsilon N\}$$

The neighborhood worst point (NWP) represents a point that is constructed from the maximum values obtained by the parent individuals’ neighborhood populations for each of the objectives. In this context, if we denote ${z(NWP)}_{i}^{*}$ as the i-th subobjective of the neighborhood worst point, its definition can be stated as follows:

(4) $${z(NWP)}_{i}^{*}=max\;\{f_{i}(x)|i=1,2,\ldots ,m,\;x\epsilon N\}$$

According to the ideal point, the neighborhood ideal point, and the neighborhood worst point, this area is partitioned into three parts. If an offspring is generated within Region III, it will be compared with the entire population and potentially replace some members of that population. When offspring are generated in Region II, the offspring will be compared with the neighbors of the parent and potentially replace some individuals within that neighborhood. When offspring are generated in Region I, the offspring will be discarded without undergoing any replacement operations.

### 3.2. MOEA/D-NRD

Algorithm 1 presents the pseudocode for MOEA/D-NRD. The algorithm is primarily divided into five main steps: initialization; solution construction; update of ideal points; neighborhood ideal points; and neighborhood worst points, selecting the appropriate reference population, and updating EP.

The algorithm commences with the provision of an input comprising a multi-objective optimization problem along with its associated solution space. The iteration count M is predefined. The variable N signifies the quantity of subproblems derived through the decomposition method; a higher N implies a greater level of detail in partitioning the multi-objective solutions space, albeit at an escalated computational cost. Each of these N subproblems corresponds to one of the N reference vectors that must be suitably configured ($\lambda^{1},\lambda^{2},\ldots {,\lambda }^{N}$).

Below are the five main steps of Algorithm 1.
**Algorithm 1:** MOEA/D-NRD**Input**: • MOP(1); • a stopping criterion; • N: the number of subproblems considered in MOEA/D; • a uniform spread of N weight vectors: $\lambda^{1}\ldots \lambda^{N}$; • T: the number of the weight vectors in the neighborhood of each weight vector, **Output: EP** 1:   Initialization; 2:   **While** not stopping criteria met **do**: 3:     Solution construction; 4:     Update ideal point, neighborhood ideal point, and neighborhood worst point; 5:     Select reference population; 6:     Update of EP; 7: **End while**

**Step 1**: Perform the initialization of the population. For each of the N subproblems indexed by i=(1,…,N), S initial solutions are randomly and uniformly generated. The weight vector z is initialized as $\vec{z}={\left(z_{1},\ldots ,z_{N}\right)}^{T}$, where $z_{i}=min\;\{f_{i}(x^{1}),\;f_{i}(x^{2}),\ldots ,\;f_{i}(x^{N})\}$. Initialize the EP as empty. Additionally, initialize the ideal points, neighborhood ideal points, and neighborhood worst points for the population.

**Step 2**: This step involves the selection of two indices k and l from a chosen population subset B(i) in a random manner. Solutions corresponding to these indices are subjected to crossover, mutation, and selection processes to generate a new solution y. Subsequently, y is refined and improved using repair heuristics or other improvement strategies to yield a modified solution y’.

**Step 3**: For each subproblem indexed by i=(1,…,N), if the newly generated solution y outperforms the current ideal point, i.e., $F_{i}(IP)<F_{i}(y)$, then set the ideal point as $F_{i}(IP)=F_{i}(y)$. If the solution is better than the current neighborhood ideal point, i.e., $F_{i}(NIP)<F_{i}(y)$, then set the neighborhood ideal point as $F_{i}(NIP)=F_{i}(y)$. If the new solution is worse than the current neighborhood worst point, i.e., $F_{i}(NWP)>F_{i}(y)$, then set the neighborhood worst point as $F_{i}(NWP)=F_{i}(y)$.

**Step 4**: The algorithm selects a suitable population for the produced solution. The possible solution domain is partitioned into three distinct areas using the ideal point, the neighborhood ideal point, and the neighborhood worst point. The suitability of the population against which the generated solution will be compared is decided based on where this solution falls within these regions. Thus, if F(NIP)≺F(y), the solution will be compared against the entire population. If F(NWP)≺F(y), the solution will only be compared against its neighborhood population. Otherwise, the offspring will be discarded.

**Step 5**: Update the EP. All vectors in the EP that are dominated by $F(y^{\prime })$ are removed. If none of the vectors in the EP dominate $F(y^{\prime })$, then $F(y^{\prime })$ is added to the EP.

Finally, through continuous iterative solving, the algorithm ultimately arrives at an approximate set of optimal solutions that closely approximates the ideal Pareto front.

The process for determining the current population type is as follows. First, assess the domination relationship between the offspring and the neighborhood ideal point. If the generated offspring dominates the neighborhood ideal point, then the population type is considered to be the entire population. Otherwise, proceed to evaluate the domination of the offspring against the neighborhood worst point. If the generated offspring dominates the neighborhood worst point, then the population type is deemed to be the neighborhood. If neither condition is met, the population type is classified as empty. The pseudocode for this procedure is illustrated as Algorithm 2.
**Algorithm 2:** Select reference population**Input**: • y: a new solution • IP: ideal point • NIP: neighborhood ideal point • NWP: neighborhood worst point **Output**: **population type** 1: if   F(NIP)<F(y) then population type = population         end for 2: else if   F(NWP)<F(y) then population type = neighborh         end for 3: else population type = none         end for

**Figure 3:**
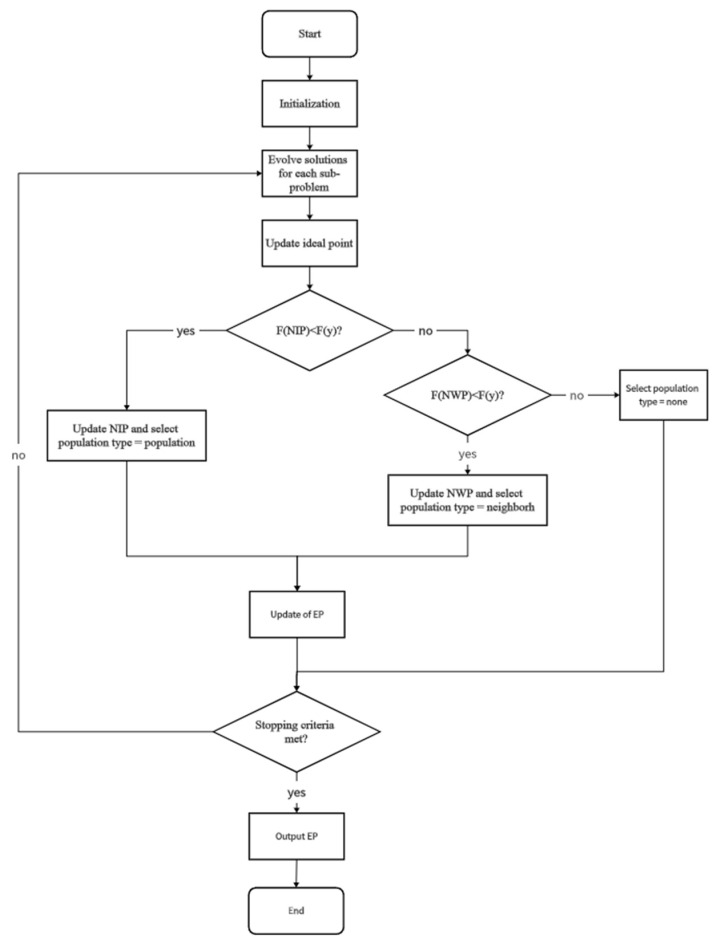
MOEA/D-NRD algorithm flowchart.

The complete MOEA/D-NRD algorithm process is illustrated through a flowchart, as shown in Figure 3. In this figure, NIP represents the neighborhood ideal point, while NWP stands for the neighborhood worst point.

Assuming the population size is N, the number of objective functions is m, and the neighborhood size for each subproblem is T. During the initialization process of MOEA/D, it needs to initialize the entire population O (N), weight vectors O (mN), and neighborhoods O $\left(mN^{2}\right)$; thus, the initialization time complexity is O $\left(mN^{2}\right)$. For MOEA/D-NRD, initialization requires additional computation of neighborhood ideal points and worst points; therefore, the initialization time complexity is O (mN(N+T)). In each iteration, MOEA/D is required to update N solutions and directly update the T neighborhood solutions associated with each solution, so the time complexity of MOEA/D is O (mNT). For MOEA/D-NRD, the update operation is consistent with that of MOEA/D, but it also needs to update the neighborhood ideal points and worst points. However, due to the setting of the number of replacements, the time complexity remains O (mNT), regardless of which individuals are chosen for replacement. The space complexity for MOEA/D involves storing population information O (mN), weight vectors O (mN), neighborhood space O (NT), and ideal points O (1), resulting in a total space complexity of O ((m+T)N). For MOEA/D-NRD, storing neighborhood ideal points and worst points introduces an additional space complexity of O (N), leading to a total space complexity of O ((m+T)N). Since T<N, it can be said that compared to the basic MOEA/D algorithm, the improved MOEA/D-NRD does not show significant changes in terms of time or space complexity.

## 4. Results

### 4.1. Comparison Function and Test Function

To validate the effectiveness of the MOEA/D-NRD algorithm based on neighborhood region domination in addressing multi-objective optimization problems, this paper employs standard benchmark test functions for multi-objective optimization. The performance of MOEA/D-NRD is compared against the original MOEA/D algorithm and its improved variants.

Given that solutions to multi-objective optimization problems typically manifest as a set of optimal trade-off solutions rather than a single solution, a common practice is to preset an ideal Pareto frontier and assess the proximity of the obtained solution set to this ideal. The closer the attained solution set is to the ideal Pareto frontier, the higher the quality of the solutions generated by the algorithm. This proximity serves as a measure to evaluate and compare the efficiency and effectiveness of different algorithms in solving these complex multi-objective problems.

This paper selects four ZDT functions [22] as the benchmark test functions for this study, and their functional expressions are presented in Table 1.

Since the DTLZ [23] test functions are all triobjective optimization problems, the DTLZ set is chosen as the test function suite for triobjective optimization by designating its number of objectives as three. Let k denote a parameter that is manually set; the expression of these functions can be found in Table 2.

### 4.2. Performance Metrics

In this paper, two widely adopted performance metrics for evaluating multi-objective optimization algorithms are employed: the inverted generational distance (IGD) metric and the hypervolume (HV) indicator.

(5) $$IGD(P^{*},P)=\frac{\sum_{v\epsilon P^{*}}d(v,P)}{\left|P^{*}\right|}$$

The IGD value represents the minimum distance from the population obtained by an algorithm to the ideal Pareto front. A smaller IGD value indicates that the population is closer to the ideal Pareto front and is distributed across all parts of it, thereby signifying better algorithmic performance. Thus, the IGD value can effectively evaluate the convergence and diversity of the algorithm.

Here, d(v,P) represents the minimum Euclidean distance from point v to P, where point P denotes the solution set obtained by the algorithm, which is a collection of solutions in the population. $P^{*}$ refers to a uniformly distributed set of points taken from the ideal Pareto front. The size of this reference set $P^{*}$ is denoted as $\left|P^{*}\right|$.

(6) $$HV(X,P)=\overset{X}{\underset{x\epsilon X}{⋃}}v(x,P)$$

The HV value is used to comprehensively evaluate both the convergence and diversity of solutions obtained by a multi-objective optimization algorithm. A larger HV value indicates that the current population is considered to be closer to the ideal Pareto front. However, the choice of reference point must be made with great caution, as it significantly impacts the accuracy of the HV metric.

v(x,P) represents the hypervolume of the space formed between solution x in the nondominated solution set *X* and reference point *P*. This is defined as the volume of a hypercube with the line segment connecting solution *x* to reference point *P* acting as one of its diagonals.

The Wilcoxon rank-sum test is a nonparametric statistical method used to investigate whether there are differences between related samples. In this study, the Wilcoxon rank-sum test is employed, and to present the comparative results between algorithms more intuitively, a simplified notation is adopted.

“+” implies that Algorithm A may exhibit significantly better performance than Algorithm B on the given test problem.

“−” suggests that Algorithm A may demonstrate significantly inferior performance compared to Algorithm B on the tested problem.

“≈” indicates that there may be no statistically significant difference in performance between Algorithm A and Algorithm B.

For this paper, the significance level is set at 0.05.

### 4.3. Test Results

For a consistent test setup, the population size is set to be the same for all problems. For each test problem, the population size is configured as 500. The neighborhood size is uniformly established at 50 across all experiments. Each algorithm is subjected to 25 independent replicate runs for each test problem. The maximum function evaluation number is employed as the termination criterion, which is set to 40,000 for all algorithms and problems. The algorithm presented herein is implemented based on the jMetal framework, utilizing the Java programming language.

**Figure 4:**
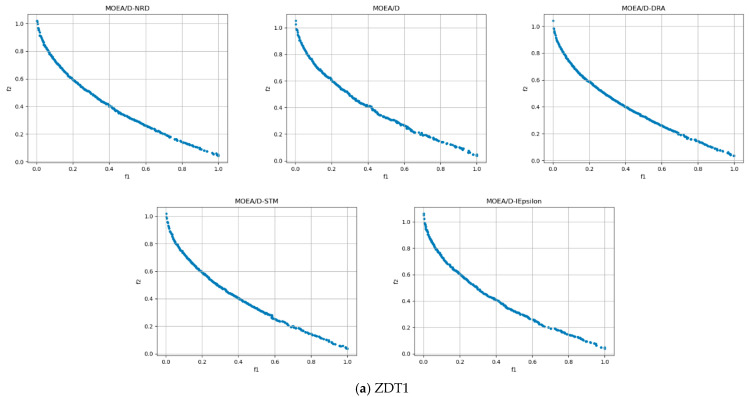

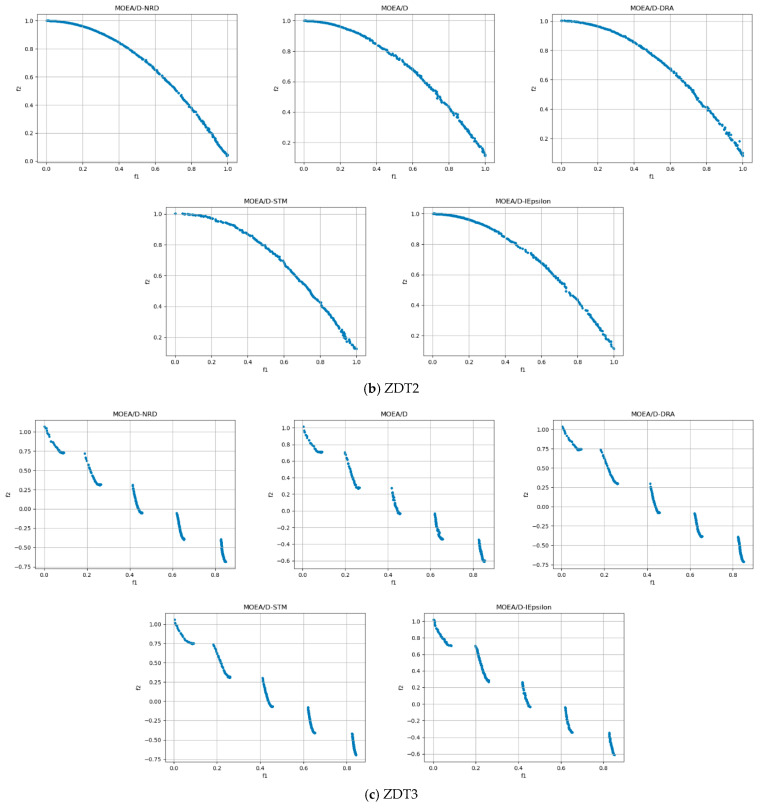

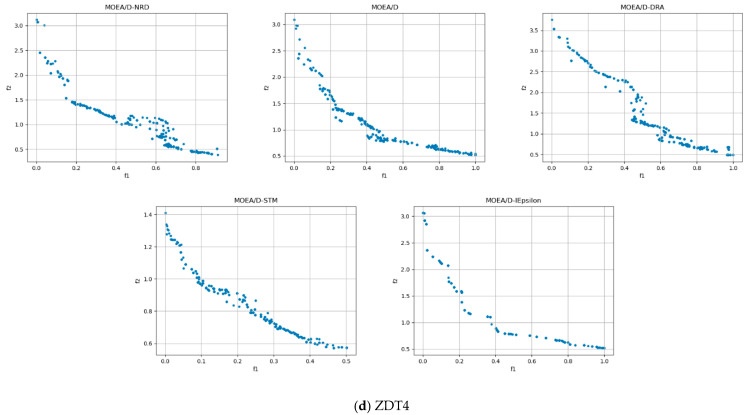
Results of five algorithms for the ZDT problem.

**Figure 5:**
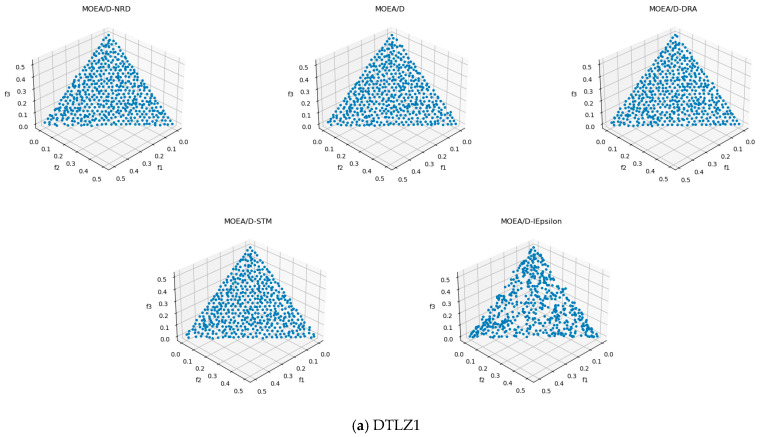

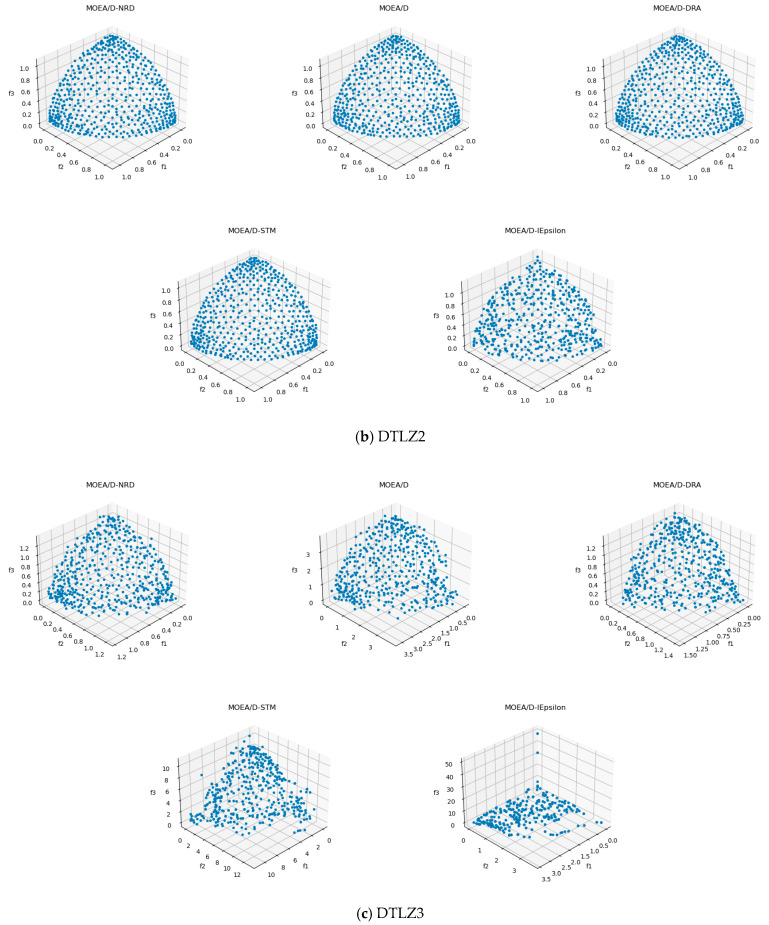

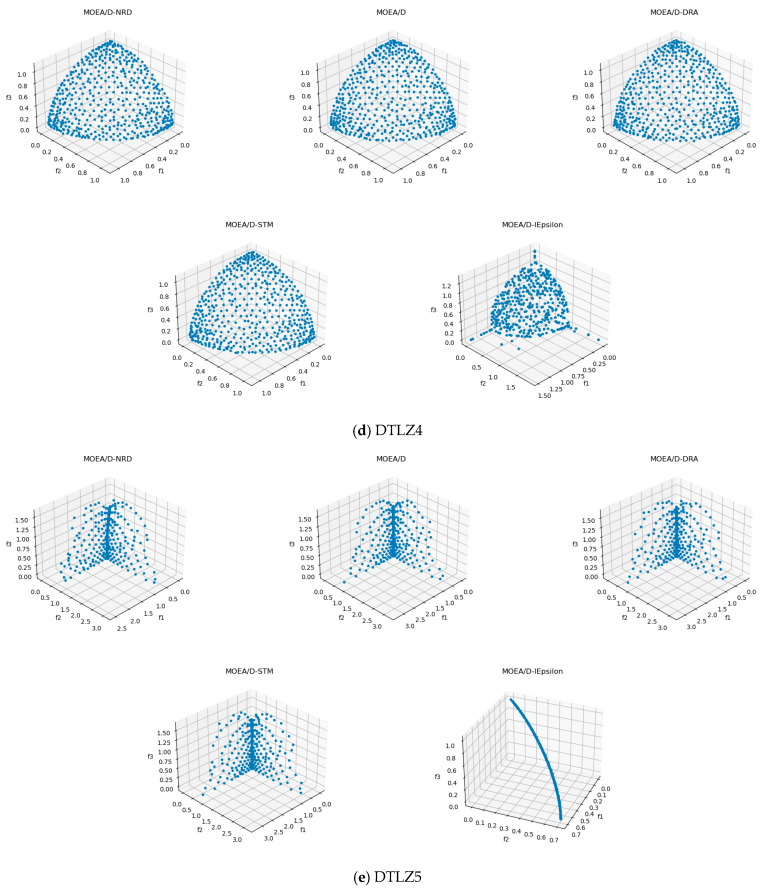

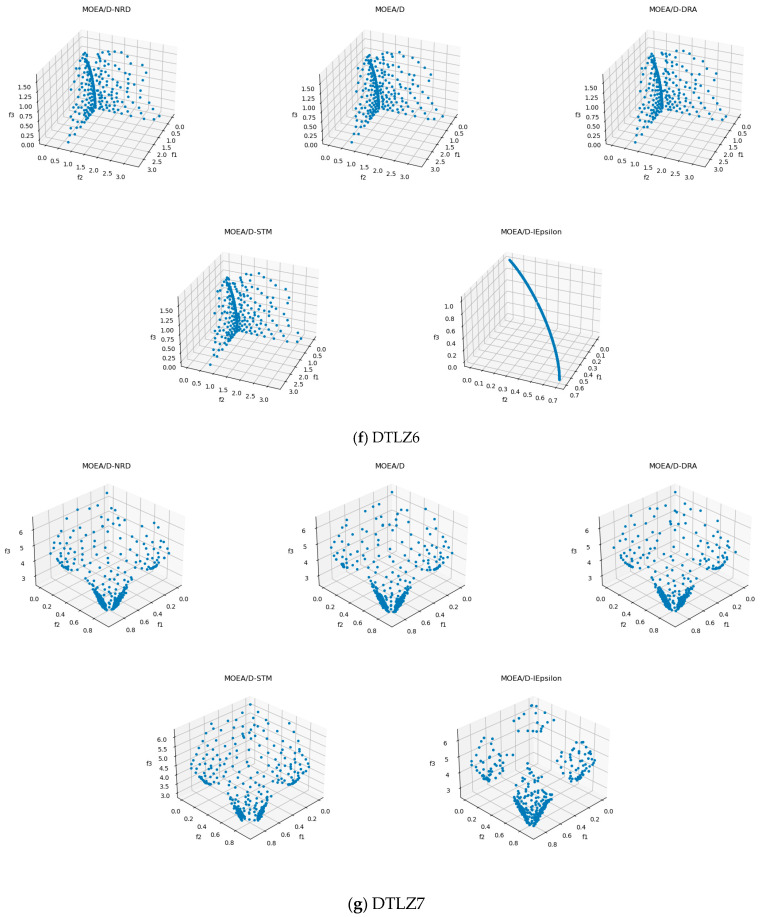
Results of five algorithms for the DTLZ problem.

In order to assess the quality of MOEA/D-NRD, it is compared with the original MOEA/D algorithm and several MOEA/D variants, MOEA/D-DRA, MOEA/D-STM, and MOEA/D-IEpsilon, in solving different multi-objective problems. Figure 4 and Figure 5, respectively, present the distribution of solutions obtained by these four algorithms when tackling the ZDT series and DTLZ series benchmark test problems.

In the ZDT1, ZDT2, and ZDT3 test sets, these five algorithms show good results overall. In the ZDT1 test set, MOEA/D-NRD exhibits excellent performance in terms of convergence, with the solution set being tightly and evenly distributed along the Pareto front, particularly standing out at the edges. MOEA/D-DRA also shows good convergence, with an overall even distribution, although it is slightly sparser at the edges of the Pareto front. In comparison, the other three algorithms (MOEA/D, MOEA/D-STM, MOEA/D-IEpsilon) exhibit some fluctuations in the distribution of the solution set and have poorer convergence.

In the ZDT2 test set, MOEA/D-NRD still performs best, with solutions more closely approaching the Pareto front and especially dense at the edges. The other algorithms are sparser at the edges, with uneven distributions and noticeable fluctuations.

In the ZDT3 test set, MOEA/D-NRD continues to excel in overall convergence, but in the first local region, the distribution of the solution set is not as uniform as that of the other algorithms, appearing especially sparse in the first two regions.

However, on the ZDT4 test set, none of the five algorithms can ideally approach the Pareto front. The solution set of MOEA/D-IEpsilon is the most sparse, with poor diversity. Nevertheless, MOEA/D-NRD still excels in convergence speed, with the tail region’s solution set closer to the Pareto front.

In the ZDT test suites, when the population evolves into boundary regions, the contrast between the neighborhood ideal point and the neighborhood worst point becomes more pronounced, making the quality assessment of solutions more accurate and promoting the formation of stable solution clusters in these regions. During the evolutionary process, in the initial stage, a large number of solutions are allocated to the global population to promote extensive exploration. In the middle stage, more solutions are assigned to the neighborhoods to enhance the local search. However, when dealing with discontinuous problems, the calculation of the neighborhood ideal point and the neighborhood worst point may be affected by breakpoints, leading to unstable criteria for solution evaluation. This explains why sparse distribution occurs in the first region of ZDT3.

In the DTLZ1, DTLZ2, and DTLZ4 test sets, MOEA/D-NRD, MOEA/D, MOEA/D-DRA, and MOEA/D-STM all exhibit good convergence, distribution, and solution-set diversity, effectively approaching the Pareto front. However, the MOEA/D-IEpsilon algorithm performs relatively poorly in these test sets, with poor diversity in the solution set, especially showing sparser density in certain regions.

In the DTLZ3 test set, MOEA/D-NRD and MOEA/D-DRA stand out, with MOEA/D-NRD demonstrating a more uniform distribution of solutions and the best convergence. In contrast, MOEA/D-DRA shows slightly sparser solutions in some extreme areas. The other algorithms exhibit greater fluctuations in their solution sets and fail to show ideal convergence under the same conditions.

For the DTLZ5 and DTLZ6 test sets, the MOEA/D-IEpsilon algorithm demonstrates the best convergence, successfully approaching the Pareto front, while the other four algorithms lack sufficient convergence, resulting in less-satisfactory overall performance.

In the DTLZ7 test set, all five algorithms show clustering of solution sets, but MOEA/D-STM performs the best, with lower levels of clustering and a more even distribution. MOEA/D-IEpsilon, on the other hand, exhibits clear segmentation in four local regions, indicating its capability to handle local optimal regions. Overall, MOEA/D-STM shows a balanced performance in the DTLZ7 test set, with good convergence and diversity.

In the DTLZ test suites, the strategy of MOEA/D-NRD is particularly evident in DTLZ3, where multiple local Pareto fronts exist. The neighborhood region domination strategy helps the algorithm avoid local optima and maintain stable convergence toward the true Pareto front. However, when encountering degenerate problems (DTLZ5-6), the dimensionality reduction of the Pareto front can undermine the effectiveness of this strategy. In low-dimensional manifolds, the distinction between the neighborhood ideal point and the worst point becomes less meaningful.

Table 3 and Table 4, respectively, display the mean, standard deviation, median, and quartiles of the IGD values obtained by the five algorithms. Statistical analysis of the experimental results using the Wilcoxon rank-sum test is conducted in Table 3. In the respective result tables, “+”, “−”, and “≈” denote that the MOEA/D-NRD algorithm outperforms, underperforms, or performs comparably to the respective algorithms. By combining the data from Table 3 and Table 4, the MOEA/D-NRD algorithm achieves better mean and median results on the ZDT test suites compared to the MOEA/D algorithm. Except for the ZDT1 test suite, where the mean IGD is slightly higher than that of the MOEA/D-DRA algorithm, the MOEA/D-NRD algorithm attains the best mean IGD values across the remaining test suites.

In the DTLZ test suites, the MOEA/D-NRD algorithm registers improvements in the form of lower, superior mean IGD values compared to the MOEA/D algorithm, achieving the best results among the five algorithms for DTLZ1 and DTLZ3. Regarding the median IGD, MOEA/D-NRD slightly surpasses MOEA/D in DTLZ4 and yields comparable outcomes in DTLZ6 while excelling in the remainder of the DTLZ test suites. Except for MOEA/D-IEpsilon, which achieves superior IGD results in DTLZ5 and DTLZ6 among these five algorithms, MOEA/D-NRD consistently exhibits the competitiveness of its approach in all other instances.

Moreover, the results from the Wilcoxon rank-sum test indicate that MOEA/D-NRD is generally comparable to MOEA/D and other MOEA/D variants across most test suites. Specifically, MOEA/D-NRD demonstrates a pronounced advantage on the ZDT2 test suite.

**Figure 6:**
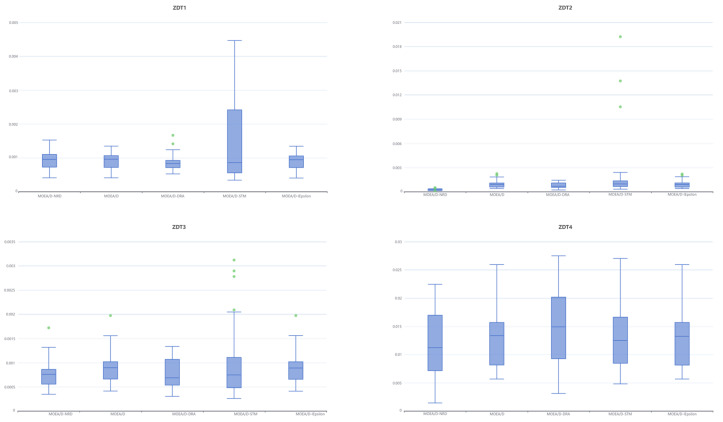
Box plots of the IGDs of algorithms in ZDT test suites.

From Figure 6’s box plots, MOEA/D-NRD achieves the best IGD values on ZDT2 and ZDT4. MOEA/D-NRD demonstrates good stability across most of the test problems, with its performance being particularly outstanding on the ZDT2 problem. MOEA/D shows moderate performance. While MOEA/D-DRA excels on certain problems (such as ZDT3), it exhibits significant fluctuations on more complex problems (like ZDT4). Although MOEA/D-STM may achieve good optimal solutions, it has the poorest overall stability and frequently produces outliers. MOEA/D-IEpsilon, on the other hand, shows a level of stability and performance that is close to that of MOEA/D-NRD.

**Figure 7:**
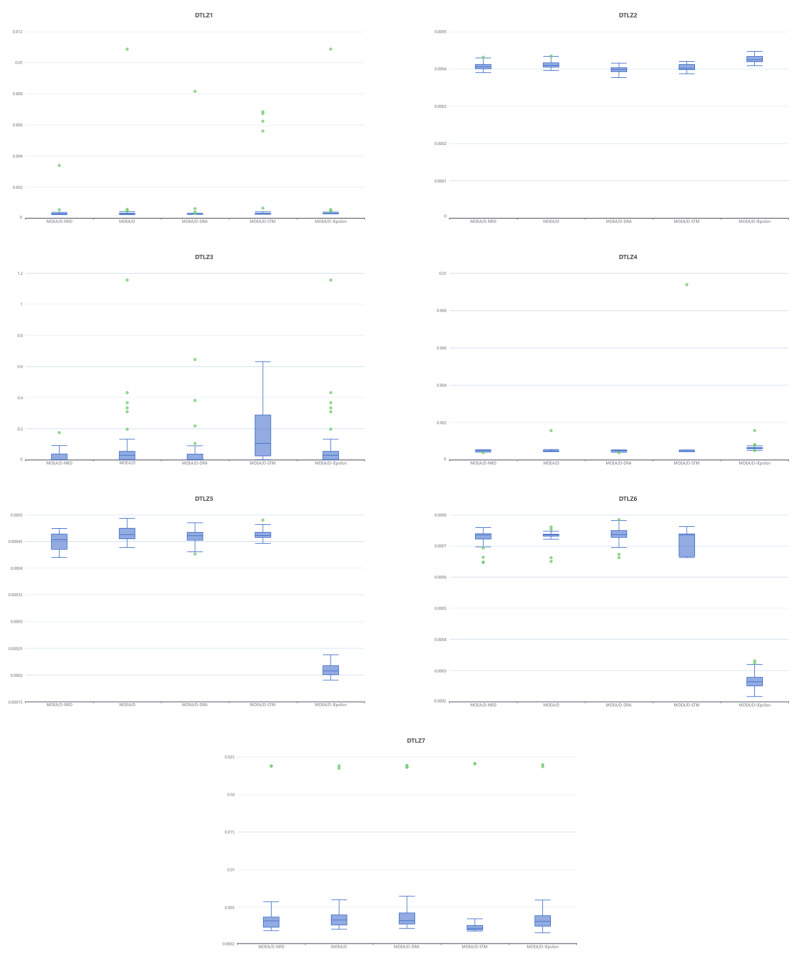
Box plots of the IGDs of algorithms in DTLZ test suites.

The stability and consistency of the MOEA/D-NRD algorithm are further underscored by the box plots presented in Figure 7. On DTLZ1 and DTLZ4, all algorithms exhibit extremely high stability and similarly excellent performance. On DTLZ2, MOEA/D-DRA shows the highest stability, while MOEA/D-IEpsilon, despite having larger fluctuations, has a better median performance. DTLZ3 is the most challenging problem, with all algorithms showing a significant number of outliers; among them, MOEA/D-STM exhibits the largest fluctuations, whereas MOEA/D-NRD and MOEA/D-DRA are relatively more stable. On DTLZ5 and DTLZ6, MOEA/D-IEpsilon demonstrates notably superior performance compared to the other algorithms. For DTLZ7, MOEA/D-STM performs the best.

In the DTLZ test suites, MOEA/D-IEpsilon stands out in specific problems, while MOEA/D-STM, although excelling in some individual cases, suffers from poor overall stability. MOEA/D-NRD, MOEA/D, and MOEA/D-DRA, on the other hand, show a more balanced and stable overall performance, with MOEA/D-NRD’s box-plot distribution being more concentrated, indicating more reliable results.

Table 5 and Table 6 present the means, standard deviations, medians, and quartiles of the HV values achieved by the five algorithms. In Table 5, the last row includes the results of a Wilcoxon rank-sum test for statistical analysis of the experimental outcomes. MOEA/D-NRD attains the highest HV mean values in ZDT2, ZDT4, DTLZ1, and DTLZ3 test suites, yet it only attains the best HV medians in ZDT2 and DTLZ3.

MOEA/D-NRD achieves the optimal IGD mean in Table 3, but its HV mean is lower than that of MOEA/D-STM. While the MOEA/D-NRD algorithm indeed shows improvement over MOEA/D in terms of the HV mean, this enhancement is not notably evident in test cases like ZDT1, DTLZ2, DTLZ5, and DTLZ6, resulting in HV medians that are roughly equivalent to those of MOEA/D. The Wilcoxon rank-sum test indicates that MOEA/D-NRD maintains the competitiveness of the original MOEA/D and even outperforms it in three test suites. It also demonstrates competitive capability against other MOEA/D variants.

**Figure 8:**
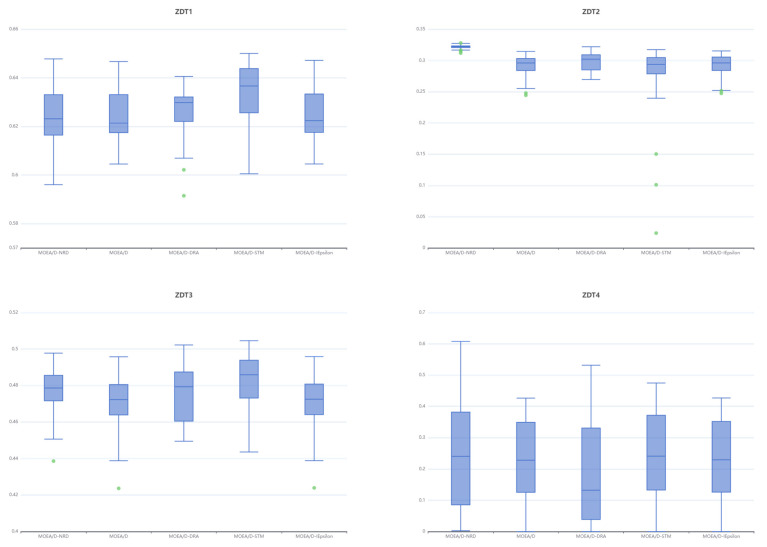
Box plots of the HV of algorithms in ZDT test suites.

From the box plots in Figure 8, it can be observed that MOEA/D-NRD exhibits strong stability and excellent solution-set distribution. Compared to other algorithms, MOEA/D-NRD shows a smaller range of fluctuations across all test sets, particularly on ZDT1, ZDT2, and ZDT3, where the compactness of the boxes indicates very good uniformity and convergence of the solution set. Unlike MOEA/D-STM and MOEA/D-IEpsilon, which show larger fluctuations and more extreme values, MOEA/D-NRD does not have noticeable outliers, demonstrating more reliable performance. Additionally, on the ZDT2 test set, MOEA/D-NRD has a leading advantage and performs the best. MOEA/D shows the most concentrated HV value, while MOEA/D-DRA and MOEA/D-STM display larger fluctuations, indicating their less stable performance on this problem.

**Figure 9:**
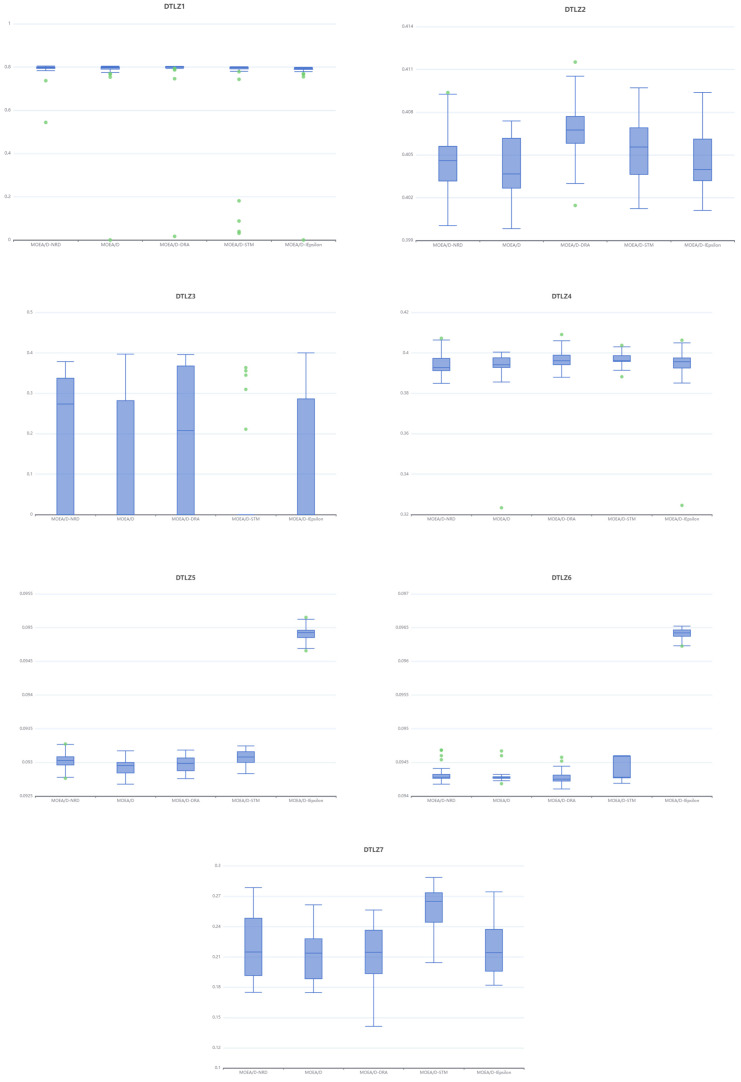
Box plots of the HV of algorithms in DTLZ test suites.

In the box plots of Figure 9, in the DTLZ1 test set, all five algorithms achieve similar HV (hypervolume) values, but MOEA/D-NRD has fewer outliers and a smaller range of fluctuations. Secondly, on the DTLZ3 test set, the performance distribution of MOEA/D-NRD is more concentrated compared to MOEA/D and MOEA/D-DRA. In the DTLZ5 and DTLZ6 test sets, MOEA/D-IEpsilon performs the best, while the other four algorithms show poorer performance. On the DTLZ7 test set, MOEA/D-STM outperforms the other algorithms, and among the remaining algorithms, MOEA/D-NRD has a higher upper quartile, indicating an advantage in finding high-quality solutions.

Across all DTLZ test sets, MOEA/D-NRD achieves the best mean HV values only for DTLZ1 and DTLZ3. In the other test sets, MOEA/D-NRD’s box plots are wider, indicating less stability in HV. Compared to other MOEA/D variants, there is still room for improvement in maintaining population diversity with MOEA/D-NRD.

Table 7 presents the computation times for the five algorithms across ZDT and DTLZ problems. The data reveal that the original MOEA/D algorithm already exhibits a certain advantage in terms of computational speed. Compared to MOEA/D, MOEA/D-NRD necessitates the initialization of neighborhood ideal points and neighborhood worst points; however, during population updating, it discards offspring worse than the worst point in the neighborhood, which somewhat reduces the running time. Both MOEA/D-NRD and MOEA/D-DRA have improved upon MOEA/D without causing a significant increase in runtime. Conversely, the enhancements introduced in MOEA/D-STM and MOEA/D-IEpsilon algorithms lead to a marked rise in computation times.

## 5. Conclusions

This paper proposes an algorithm based on neighborhood region domination, MOEA/D-NRD, for solving multi-objective optimization problems based on neighborhood reference domination. In MOEA/D-NRD, during the population update process, offspring solutions are evaluated against their respective neighborhood ideal points and neighborhood worst points to determine domination relationships. By assessing the domination relationship between offspring solutions and their respective neighborhood ideal points as well as neighborhood worst points, MOEA/D-NRD selects appropriate solution-set comparison regions. MOEA/D-NRD exploits superior solutions more while reducing the use of inferior ones, a process that enables a larger portion of the solutions to converge faster toward the ideal point, thereby accelerating the population convergence and enhancing the computational efficiency of the algorithm. The performance of the solutions generated by this MOEA/D-NRD algorithm surpasses those of the original MOEA/D, and it outperforms other MOEA/D variants in certain test suites.

The MOEA/D-NRD algorithm introduced in this paper has proven effective in enhancing the quality of solutions for biobjective optimization problems. Nonetheless, the MOEA/D-NRD algorithm still has room for improvement in solving triobjective problems, and there is still scope for improvement in maintaining population diversity. Consequently, enhancing the performance of the MOEA/D-NRD algorithm and boosting the diversity of its solutions constitute vital avenues for future research, aiming to ensure good performance in tackling multi-objective problems of even higher dimensionalities.

## Figures and Tables

**Table 1:** ZDT test function table.

Problem	Objective Functions	Variable Bounds
ZDT1	$Min\;F=\{f_{1},\;f_{2}\}$ $f_{1}=x_{1}$ $f_{2}=g\times h$ $g=1+9\times \sum_{i=2}^{n}x_{i}/(n-1)$ $h(f_{1},g)=1-\sqrt{f_{1}/g}$	n=30 $x_{i}\epsilon \;[0,\;1]$
ZDT2	$Min\;F=\{f_{1},\;f_{2}\}$ $f_{1}=x_{1}$ $f_{2}=g\times h$ $g=1+9\times \sum_{i=2}^{n}x_{i}/(n-1)$ $h(f_{1},g)=1-{\left(f_{1}/g\right)}^{2}$	n=30 $x_{i}\epsilon \;[0,\;1]$
ZDT3	$Min\;F=\{f_{1},\;f_{2}\}$ $f_{1}=x_{1}$ $f_{2}=g\times h$ $g=1+9\times \sum_{i=2}^{n}x_{i}/(n-1)$ $h(f_{1},g)=1-\sqrt{f_{1}/g}-(f_{1}/g)\times sin(10\pi f_{1})$	n=30 $x_{i}\epsilon \;[0,\;1]$
ZDT4	$Min\;F=\{f_{1},\;f_{2}\}$ $f_{1}=x_{1}$ $f_{2}=g\times h$ g=1+10×(n−1) $+\sum_{i=2}^{n}\left(x_{i}^{2}-10cos(4\pi x_{i})\right)$ $h(f_{1},g)=1-\sqrt{f_{1}/g}$	n=10 $x_{1}\epsilon \;[0,\;1]$ $x_{2},...,x_{n}\epsilon \;[-5,\;5]$

**Table 2:** DTLZ test function table.

Problem	Objective Functions	Variable Bounds
DTLZ1	$Min\;F=\{f_{1},f_{2},f_{3}\}$ $f_{1}=(1+g)*0.5*x_{1}x_{2}$ $f_{2}=(1+g)*0.5*x_{1}(1-x_{2})$ $f_{3}=(1+g)*0.5*{(1-x}_{1})$ $g=100\;[|x_{m}|+\sum_{x_{i}\in x_{M}}{\left(x_{i}-0.5\right)}^{2}-cos(20\pi (x_{i}-0.5))]$	$x_{i}\epsilon \;[0,\;1]$
DTLZ2	$Min\;F=\{f_{1},f_{2},f_{3}\}$ $f_{1}=(1+g)cos(x_{1}\pi /2)cos(x_{2}\pi /2)$ $f_{2}=(1+g)cos(x_{1}\pi /2)sin(x_{2}\pi /2)$ $f_{3}=(1+g)sin(x_{1}\pi /2)$ $g=\sum_{x_{i}\in x_{M}}{\left(x_{i}-0.5\right)}^{2}$	$x_{i}\epsilon \;[0,\;1]$
DTLZ3	$Min\;F=\{f_{1},f_{2},f_{3}\}$ $f_{1}=(1+g)cos(x_{1}\pi /2)cos(x_{2}\pi /2)$ $f_{2}=(1+g)cos(x_{1}\pi /2)sin(x_{1}\pi /2)$ $f_{3}=(1+g)sin(x_{1}\pi /2)$ $g=100\;[|x_{m}|+\sum_{x_{i}\in x_{M}}{\left(x_{i}-0.5\right)}^{2}-cos(20\pi (x_{i}-0.5))]$	$x_{i}\epsilon \;[0,\;1]$
DTLZ4	$Min\;F=\{f_{1},f_{2},f_{3}\}$ $f_{1}=(1+g)cos(x_{1}^{\alpha }\pi /2)cos(x_{2}^{\alpha }\pi /2)$ $f_{2}=(1+g)cos(x_{1}^{\alpha }\pi /2)sin(x_{2}^{\alpha }\pi /2)$ $f_{3}=(1+g)sin(x_{1}^{\alpha }\pi /2)$ $g=\sum_{x_{i}\in x_{M}}{\left(x_{i}-0.5\right)}^{2}$	$x_{i}\epsilon \;[0,\;1]$
DTLZ5	$Min\;F=\{f_{1},f_{2},f_{3}\}$ $f_{1}=(1+g)cos(\theta_{1}\pi /2)cos(\theta_{2}\pi /2)$ $f_{2}=(1+g)cos(\theta_{1}\pi /2)sin(\theta_{2}\pi /2)$ $f_{3}=(1+g)sin(\theta_{1}\pi /2)$ $\theta_{i}=\frac{\pi }{4(1+g)}(1+2gx_{i})$ $g=\sum_{x_{i}\in x_{M}}{\left(x_{i}-0.5\right)}^{2}$	$x_{i}\epsilon \;[0,\;1]$
DTLZ6	$Min\;F=\{f_{1},f_{2},f_{3}\}$ $f_{1}=(1+g)cos(\theta_{1}\pi /2)cos(\theta_{2}\pi /2)$ $f_{2}=(1+g)cos(\theta_{1}\pi /2)sin(\theta_{2}\pi /2)$ $f_{3}=(1+g)sin(\theta_{1}\pi /2)$ $\theta_{i}=\frac{\pi }{4(1+g)}(1+2gx_{i})$ $g=\sum_{x_{i}\in x_{M}}x_{i}^{0.1}$	$x_{i}\epsilon \;[0,\;1]$
DTLZ7	$Min\;F=\{f_{1},f_{2},f_{3}\}$ $f_{1}=x_{1}$ $f_{2}=x_{2}$ $f_{3}=x_{3}$	$x_{i}\epsilon \;[0,\;1]$

**Table 3:** IGD means and standard deviations of algorithms.

	MOEA/D-NRD	MOEA/D	MOEA/D-DRA	MOEA/D-STM	MOEA/D-IEpsilon
ZDT1	9.05E-4(2.70E-4)	9.21E-4(2.42E-4)	**8.72E-4(2.46E-4)**	1.45E-3(1.25E-3)	9.14E-4(2.42E-4)
ZDT2	**2.50E** **-4** **(8.79E-5** **)**	9.81E-4(4.70E-4)	7.40E-4(3.67E-4)	2.55E-3(4.60E-3)	9.73E-4(4.66E-4)
ZDT3	**7.62E-4(2.84E** **-4)**	8.82E-4(3.05E-4)	8.05E-4(3.14E-4)	1.01E-3(8.14E-4)	8.79E-4(3.05E-4)
ZDT4	**1.20E** **-2(** **6.11E-** **3)**	1.30E-2(5.51E-3)	1.45E-2(6.74E-3)	1.32E-2(5.48E-3)	1.30E-2(5.53E-3)
DTLZ1	**4.12E-4(6.10E-4)**	7.23E-4(2.07E-3)	5.95E-4(1.54E-3)	1.26E-3(2.23E-3)	7.44E-4(2.07E-3)
DTLZ2	4.07E-4(9.44E-6)	4.10E-4(9.56E-6)	**3.97E** **-4(** **9.00E-6** **)**	4.04E-4(9.16E-6)	4.26E-4(9.04E-6)
DTLZ3	**2.** **17E-2(3.92E-2** **)**	1.25E-1(2.47E-1)	6.24E-2(1.46E-1)	1.92E-1(2.01E-1)	1.25E-1(2.47E-1)
DTLZ4	4.77E-4(3.10E-5)	5.20E-4(2.15E-4)	**4.74E-4(** **3** **.47E-5** **)**	8.23E-4(1.75E-3)	6.59E-4(1.99E-4)
DTLZ5	4.51E-4(1.56E-5)	4.64E-4(1.30E-5)	4.60E-4(1.26E-5)	4.63E-4(8.83E-6)	**2.09E-4(1.17E-5)**
DTLZ6	7.24E-4(3.12E-5)	7.31E-4(2.32E-5)	7.38E-4(2.59E-5)	7.18E-4(3.41E-5)	**2.65E** **-4** **(2.59E-5** **)**
DTLZ7	4.66E-3(5.68E-3)	4.85E-3(5.58E-3)	6.46E-3(7.56E-3)	**4.00E-3** **(5.** **93E** **-3)**	4.69E-3(5.68E-3)
+/−/≈	-	3/0/8	2/1/8	3/1/7	5/2/4

Boldface indicates the best results in each test set.

**Table 4:** Medians and interquartile ranges of IGDs for the algorithms.

	MOEA/D-NRD	MOEA/D	MOEA/D-DRA	MOEA/D-STM	MOEA/D-IEpsilon
ZDT1	9.52E-4(7.25E-4)	9.60E-4(7.18E-4)	**8.29E-4(7.11E-4)**	8.57E-4(5.55E-4)	9.43E-4(7.13E-4)
ZDT2	**2.35E-4(2.14E** **-4)**	8.97E-4(5.78E-4)	6.79E-4(5.30E-4)	9.62E-4(6.43E-4)	8.86E-4(5.71E-4)
ZDT3	7.58E-4(5.56E-4)	8.94E-4(6.60E-4)	**6.86E-4(** **5.** **36E** **-4)**	7.46E-4(4.81E-4)	8.90E-4(6.57E-4)
ZDT4	**1.12E-** **2** **(7.15E-** **3)**	1.34E-2(8.15E-3)	1.49E-2(9.27E-3)	1.25E-2(8.46E-3)	1.32E-2(8.14E-3)
DTLZ1	2.78E-4(2.58E-4)	2.78E-4(2.51E-4)	**2** **.56E-** **4** **(** **2** **.49E-4** **)**	2.72E-4(2.59E-4)	3.01E-4(2.83E-4)
DTLZ2	4.05E-4(4.00E-4)	4.09E-4(4.05E-4)	**3.98E** **-4(** **3.92E-4** **)**	4.02E-4(3.98E-4)	4.24E-4(4.19E-4)
DTLZ3	**1.86E** **-3(** **1.19E-3** **)**	2.87E-2(1.70E-3)	2.35E-3(9.63E-4)	1.05E-1(2.48E-2)	2.85E-2(1.67E-3)
DTLZ4	4.77E-4(4.63E-4)	4.73E-4(4.59E-4)	4.79E-4(4.57E-4)	**4.59E-4(4.51E** **-4)**	6.26E-4(5.95E-4)
DTLZ5	4.54E-4(4.35E-4)	4.63E-4(4.55E-4)	4.61E-4(4.53E-4)	4.61E-4(4.58E-4)	**2.08E** **-4(** **2.01E** **-4)**
DTLZ6	7.36E-4(7.22E-4)	7.36E-4(7.32E-4)	7.36E-4(7.28E-4)	7.36E-4(6.67E-4)	**2.64E** **-4(** **2.51E** **-4)**
DTLZ7	3.19E-3(2.36E-3)	3.31E-3(2.65E-3)	3.23E-3(2.77E-3)	**2.** **20E-3(** **2** **.00E** **-3)**	3.14E-3(2.49E-3)

Boldface indicates the best results in each test set.

**Table 5:** HV medians and quartiles of algorithms.

	MOEA/D-NRD	MOEA/D	MOEA/D-DRA	MOEA/D-STM	MOEA/D-IEpsilon
ZDT1	6.25E-1(1.23E-2)	6.24E-1(1.11E-2)	6.26E-1(1.11E-2)	**6.32E** **-1(** **1.34E** **-2)**	6.25E-1(1.11E-2)
ZDT2	**3.22E** **-1(** **3.56E-3** **)**	2.91E-1(1.78E-2)	3.00E-1(1.53E-2)	2.69E-1(6.94E-2)	2.92E-1(1.74E-2)
ZDT3	4.77E-1(1.31E-2)	4.71E-1(1.43E-2)	4.75E-1(1.53E-2)	**4.83E** **-1(** **1.54E** **-2)**	4.72E-1(1.43E-2)
ZDT4	**2** **.46E-** **1** **(1.65E** **-1)**	2.23E-1(1.28E-1)	1.99E-1(1.70E-1)	2.43E-1(1.41E-1)	2.26E-1(1.29E-1)
DTLZ1	**7.85E** **-1(** **5.07E-2** **)**	7.61E-1(1.56E-1)	7.66E-1(1.53E-1)	6.81E-1(2.61E-1)	7.58E-1(1.55E-1)
DTLZ2	4.05E-1(2.10E-3)	4.04E-1(2.12E-3)	**4** **.07E-1(1.** **97E-3** **)**	4.05E-1(2.18E-3)	4.05E-1(2.20E-3)
DTLZ3	**2.16E-** **1** **(** **1** **.54E** **-1)**	1.18E-1(1.57E-1)	1.97E-1(1.73E-1)	6.34E-2(1.29E-1)	1.20E-1(1.60E-1)
DTLZ4	3.94E-1(5.09E-3)	3.92E-1(1.44E-2)	**3.97E** **-1(** **4.23E-** **3)**	3.81E-1(7.78E-2)	3.93E-1(1.46E-2)
DTLZ5	9.30E-2(1.28E-4)	9.29E-2(1.38E-4)	9.30E-2(1.15E-4)	9.31E-2(1.06E-4)	**9.49E** **-2(1.** **08E-4** **)**
DTLZ6	9.43E-2(1.37E-4)	9.43E-2(1.02E-4)	9.43E-2(1.06E-4)	9.44E-2(1.48E-4)	**9.64E** **-2(** **7.06E-5** **)**
DTLZ7	2.20E-1(3.05E-2)	2.12E-1(2.54E-2)	2.13E-1(2.83E-2)	**2.** **57E-1(** **2** **.38E** **-2)**	2.19E-1(2.65E-2)
+/−/≈	-	3/0/8	1/2/8	2/2/7	3/2/6

Boldface indicates the best results in each test set.

**Table 6:** Medians and interquartile ranges of HV for algorithms.

	MOEA/D-NRD	MOEA/D	MOEA/D-DRA	MOEA/D-STM	MOEA/D-IEpsilon
ZDT1	6.23E-1(6.16E-1)	6.21E-1(6.17E-1)	6.30E-1(6.22E-1)	**6.37E** **-1(** **6.26E-1** **)**	6.22E-1(6.18E-1)
ZDT2	**3.22E** **-1(** **3.21E-1** **)**	2.96E-1(2.84E-1)	3.02E-1(2.85E-1)	2.94E-1(2.79E-1)	2.96E-1(2.84E-1)
ZDT3	4.79E-1(4.72E-1)	4.72E-1(4.64E-1)	4.79E-1(4.60E-1)	**4.86E** **-1(** **4.73E-1** **)**	4.72E-1(4.64E-1)
ZDT4	2.41E-1(8.54E-2)	2.28E-1(1.26E-1)	1.32E-1(3.86E-2)	**2** **.41E-** **1** **(1.33E** **-1)**	2.29E-1(1.26E-1)
DTLZ1	7.97E-1(7.93E-1)	7.97E-1(7.90E-1)	**8.00E** **-1(** **7.98E-1** **)**	7.97E-1(7.91E-1)	7.93E-1(7.89E-1)
DTLZ2	4.05E-1(4.03E-1)	4.04E-1(4.03E-1)	**4** **.07E-1(** **4.06E-** **1)**	4.06E-1(4.04E-1)	4.04E-1(4.03E-1)
DTLZ3	**2.73E** **-1(** **0.00E+0** **)**	0.00E+0(0.00E+0)	2.08E-1(0.00E+0)	0.00E+0(0.00E+0)	0.00E+0(0.00E+0)
DTLZ4	3.93E-1(3.91E-1)	3.94E-1(3.93E-1)	3.96E-1(3.94E-1)	**3.96E** **-1(** **3.96E-1** **)**	3.96E-1(3.93E-1)
DTLZ5	9.30E-2(9.30E-2)	9.30E-2(9.28E-2)	9.30E-2(9.29E-2)	9.31E-2(9.30E-2)	**9.49E** **-2(** **9.49E-2** **)**
DTLZ6	9.43E-2(9.43E-2)	9.43E-2(9.43E-2)	9.43E-2(9.42E-2)	9.43E-2(9.43E-2)	**9.** **64E** **-2(** **9.64E-2** **)**
DTLZ7	2.15E-1(1.92E-1)	2.14E-1(1.88E-1)	2.15E-1(1.94E-1)	**2.65E-** **1(2.** **44E-1** **)**	2.14E-1(1.96E-1)

Boldface indicates the best results in each test set.

**Table 7:** Average running times of algorithms (millisecond, ms).

	MOEA/D-NRD	MOEA/D	MOEA/D-DRA	MOEA/D-STM	MOEA/D-IEpsilon
ZDT1	408	400	437	7035	2675
ZDT2	652	370	397	7014	1859
ZDT3	421	406	430	6980	2042
ZDT4	395	380	390	6256	1261
DTLZ1	725	695	708	11,655	15,860
DTLZ2	733	725	734	11,991	13,656
DTLZ3	757	726	735	11,419	5170
DTLZ4	733	719	732	11,911	12,836
DTLZ5	733	727	735	11,498	9660
DTLZ6	729	723	728	11,648	10,797
DTLZ7	699	694	706	11,536	10,373

## Data Availability

The raw data supporting the conclusions of this article will be made available by the authors upon request.
